# Lipid metabolic reprogramming by traditional Chinese medicine and its role in effective cancer therapy

**DOI:** 10.7150/jca.86683

**Published:** 2023-07-09

**Authors:** Hui Liu, Xiuming Li, Yajie Dong, Changhua Zhou, Caidan Rezeng

**Affiliations:** 1 Chengde Medical University, Chengde, China, Hebei 067000, China.; 2 Department of Urology, Affiliated Hospital of Chengde Medical University, Hebei 067000, China.; 3 Department of Pediatrics, Chengde County Hospital of Traditional Chinese Medicine, Hebei 067000, China.; 4 School of Pharmacy, Qinghai University for Nationalities, Qinghai, 810000, China.; 5 Engineering Research Center for Pharmaceutics of Chinese Materia Medica and New Drug Development, Ministry of Education, Beijing 100029, China.

**Keywords:** Traditional Chinese medicine, TCM, tumor, lipid metabolism reprogramming, cancer therapy

## Abstract

Epidemiological data have shown a positive correlation between lipid levels and tumor occurrence, such as the correlation between tumor frequency and aggressiveness, and cardiovascular disease, obesity, type 2 diabetes mellitus, and hyperinsulinemia. Therefore, reducing fat accumulation or weakening lipid metabolism may affect the carcinogenic processes of cells. Many studies have shown that traditional Chinese Medicine (TCM) has obvious advantages over traditional therapies in terms of fewer side effects, lower toxicity, and lower economic burden. This paper reviews the mechanism by which TCM regulates lipid metabolism and its antitumor effect through this regulation, with the aim of elucidating the bioactive compounds in TCM with good efficacy and few side effects that can provide promising therapeutic drugs for targeting lipid metabolism reprogramming in cancer.

## Introduction

Metabolic reprogramming refers to the metabolic changes that cells undergo in response to various stressors 1. Metabolic reprogramming is a widespread phenomenon in a variety of diseases, including metabolic pathways such as glucose, lipid, and amino acid metabolism, which are closely related to the occurrence and development of diseases 2. Unlike normal cells, tumors rely primarily on glycolysis rather than mitochondrial oxidative phosphorylation for energy 3. Because the energy supply of tumor cells through the glycolytic pathway is very inefficient, the energy required by tumor cells to maintain their rapid proliferation can be provided by increasing lipid metabolism in addition to increasing glucose intake and consumption 4. Increasing epidemiological data show a positive correlation between lipid levels and tumor occurrence, such as the correlation between tumor frequency and aggressiveness, and cardiovascular disease, obesity, type 2 diabetes mellitus, and hyperinsulinemia 5. Several statins that regulate lipid levels, such as simvastatin, lovastatin, and mevastatin, have been shown to inhibit tumor growth 6-8. Therefore, reducing fat accumulation or weakening lipid metabolism may affect the carcinogenic processes of cells.

Traditional Chinese medicine (TCM) has been widely used alone or as a complementary approach for cancer treatment in East Asia for hundreds of years 9. A large amount of evidence has shown that TCM has obvious advantages over traditional therapies in terms of fewer side effects, lower toxicity, and lower economic burden 9-11. Although previous studies have summarized the progress of the antitumor effect of TCM through the regulation of metabolic pathway reprogramming 12-14, the specific mechanism by which TCM regulates lipid metabolism and mediates antitumor effects has not been systematically summarized. This paper reviews the mechanism by which TCM regulates lipid metabolism and its antitumor effect through this regulation, with the aim of elucidating the bioactive compounds in TCM with good efficacy and few side effects that can provide promising therapeutic drugs for targeting lipid metabolism reprogramming in cancer.

## Biology of lipid metabolism

### Lipid metabolism in normal cells

Lipids is the general term for triacylglycerol and lipids, which also include sterol, sterol lipids, phospholipids, and glycolipids. Lipids play an important role in the biological processes of energy supply, biofilm formation, energy storage, and generation of signaling molecules 15. Lipid metabolism refers to the emulsification of most of the fat ingested by the human body into small particles by bile, and the hydrolysis of fatty acids in fat into free fatty acids and monoglycerides (and occasionally complete hydrolysis into glycerol and fatty acids) by lipases in the pancreas and small intestine. Hydrolyzed small molecules such as glycerol and short- and medium-chain fatty acids are absorbed into the bloodstream by the small intestine. After monolipids and long-chain fatty acids are absorbed, triglycerides are first resynthesized in small intestinal cells, together with phospholipids, cholesterol, and proteins, to form chylomicrons, which enter the blood circulation from the lymphatic system 16. Fatty acids are important components of various lipids that play a vital role in cells 17. Raw materials for fatty acid synthesis are catalyzed by adenosine-triphosphate (ATP)-citrate lyase (ACLY), acetyl-CoA carboxylase (ACC), and fatty acid synthase (FASN) to synthesize fatty acids from de facto. Some fatty acids are used in the synthesis of triacylglycerols and are stored in tissues as an energy supply 18. When the body requires energy, the triacylglycerol stored in the cells is broken down into glycerol and fatty acids under the action of lipase. Glycerol is decomposed by the glycolic pathway or glucose is generated by the gluconeogenic pathway to provide energy for cells, whereas fatty acids are decomposed into acetyl- CoA under sufficient oxygen supply and are thoroughly oxidized into CO_2_ and H_2_O, releasing a large amount of energy 19. Most tissues can oxidize fatty acids, except the brain tissues, because fatty acids cannot pass through the blood-brain barrier 20. Other fatty acids are used to form biofilms and produce lipid signaling molecules that meet the needs of cell division, proliferation, and signal transduction 21. In the normal body, total fatty acids are mainly derived from exogenous fatty acids obtained from food, and the proportion of fatty acids produced through de novo synthesis is very small 22.

Cholesterol can be synthesized in almost all tissues of the body; the liver is the main site, and synthesis of cholesterol is primarily carried out in the cytosol and endoplasmic reticulum 23. Acetyl-CoA is the building block for cholesterol synthesis and 3-hydroxy-3-methylglutaryl-CoA reductase (HMGCR) is a restriction enzyme in cholesterol synthesis that is regulated by the level of cellular free cholesterol 24. Cholesterol can be converted into bile acids, sterol hormones, or 7-dehydrogenated cholesterol in the body, among which the transformation into bile acids is the main route of cholesterol metabolism 23.

### Lipid metabolism in tumor cells

#### Fatty acid metabolism

In rapidly proliferating cells, fatty acid synthesis is accelerated, providing large amounts of lipids for cell membrane components and facilitating β-oxidation of proteins and fatty acyl modification. Therefore, increased fatty acid synthesis plays an important role in highly proliferating cancer cells 25. In normal cells, exogenous fatty acids taken from food are mainly used, and de novo fatty acid synthesis is inhibited; but, the enhancement of de novo fatty acid synthesis pathway in tumor cells promotes the synthesis of tumor biofilms and increases membrane lipid saturation, thereby affecting signal transduction, gene expression, and other basic life processes 26. The enhancement of de facto fatty acid synthesis pathway is the main manifestation of lipid metabolic reprogramming in tumor cells, which involves a variety of key enzymes, including increased expression of ACLY, ACC, and FASN 27, 28. ACLY is the first key enzyme in de novo synthesis of fatty acids, which also links glycolysis and lipid metabolism pathways. Multiple studies have shown that ACLY is highly expressed in tumors, including gastric cancer 29, non-cellular lung cancer 30, breast cancer (BC) 31, and ovarian cancer 32, and is associated with poor prognosis. Further studies have found that ACLY inhibitors inhibit tumor growth, further supporting the role of ACLY in promoting cancer 33-35. Notably, miRNAs have been found to inhibit de novo lipogenesis by downregulating ACLY expression, thus inhibiting tumor growth and metastasis 36, 37. ACC is a key enzyme that catalyzes the production of acetyl-CoA and malonyl-CoA and participates in the de novo synthesis of fatty acids. ACC overexpression has been detected in early BC, ductal carcinoma in situ (DCIS), and lobular carcinoma in situ; further, the phosphorylation levels of ACC are closely associated with BC and lung cancer metastasis 38. In addition, it has been found that increased ACC expression is accompanied by increased FASN and ACLY expression in prostate and hepatocellular carcinoma 39, indicating that ACC may play a synergistic role with FASN and ACLY to promote tumor growth. Liu et al. 40 showed that ACC depletion suppresses de novo fatty acid synthesis and mitochondrial beta-oxidation in the synthesis of acetyl-Coa carboxylase castration-resistant prostate cancer cells. Moreover, many studies have found that regulating fatty acid levels by interfering with ACC expression can help inhibit tumor growth 41-43. Therefore, ACC1 inhibition has become an appealing choice for antitumor therapy 43, 44. Encouraging results indicate that some specific tumor types may respond to ACC1 inhibition. It may be one of the hot spots for future studies to expand the clinical indications of ACC1 inhibitors. ACC catalyzes the formation of malonyl-CoA and gradually synthesizes fatty acids through the action of FASN 45. FASN upregulation is a common feature of human cancers and their precancerous lesions and is closely associated with chemotherapy resistance, tumor metastasis, and poor patient prognosis 46. Overexpression of FASN in tumors is dependent on the phosphatidylinositol 3 kinase (PI3K)/protein kinase B (AKT) signal transduction pathway and the transcriptional control of the solid alcohol modulator, junction egg white. Activated PI3K/AKT activates sterol regulatory element binding protein-1c (SREBP-1c) and promotes its entry into the nucleus, thereby inducing the expression of adipose-synthesis-related genes 47. In addition, adenosine monophosphate-activated protein kinase (AMPK)/mammalian target of Rapamycin (mTOR) 48 and signal transducer and activator of transcription 3 (STAT3) 49 signaling pathways have also been reported to mediate the regulation of FASN in tumor proliferation and metastasis. It is worth noting that inhibitors of FASN, similar to the two abovementioned enzymes, also inhibit tumor progression 50-52.

In tumor cells, with fatty acid removal, enhanced head group synthesis occurs together with enhanced fatty acid oxidation (FAO) 53. Carnitine palmitoyltransferase 1 (CPT1) is a key enzyme in FAO. Fatty acids are first activated into fatty acyl coenzyme A and then transported to the mitochondria by CPT1 for FAO 54. After dehydrogenation, water addition, re-dehydrogenation, and thiohydrolysis, acetyl-coenzyme A is generated, which enters the tricarboxylic acid cycle. This process not only generates ATP to supply energy to cells, but also prevents lipid toxicity caused by excessive lipid accumulation 54. The resulting acetyl-CoA enters the cytoplasm and participates in the metabolic response to generate NADPH, which generates large amounts of NADPH to support cellular redox homeostasis, thereby preventing oxidative damage in tumor cells 55. FAO plays a key role in tumor cell proliferation and resistance to chemotherapy. Inhibition of FAO in the mitochondria can affect the production of NADPH and increase the production of reactive oxygen species, leading to ATP depletion in glioblastoma cells and cell death 56. Targeting CPT1 enhances the effect of radiotherapy in patients with nasopharyngeal carcinoma 57. In addition, studies have shown that mitochondrial FAO reprogramming is enhanced in breast cancer, and CPT1A expression is elevated in recurrent breast cancer, which is associated with poor prognosis in patients with breast cancer 58. The de facto fatty acid synthesis pathway is enhanced in tumor cells, and a large number of synthesized fatty acids can supply energy to tumor cells, while FAO is also significantly enhanced 59. Both are in dynamic equilibrium to a certain extent, under which FAO provides energy, and lipid toxicity caused by excessive accumulation of fatty acids is prevented, creating favorable conditions for tumor progression.

#### Cholesterol metabolism

Abnormal activation of the cholesterol anabolic pathway is one of the signs of several tumors, which helps in the rapid growth of tumor cells to synthesize cell membranes, required lipids, and conduct necessary signals. It is characterized by the activation of cholesterol synthesis signaling SREBP-1 60 and the inhibition of cholesterol efflux signaling liver X receptors (LXRs) 61. The activation of SREBP-1 is regulated by negative feedback of intracellular cholesterol concentration, but tumor cells can bypass this regulation in several ways, allowing continued activation. Normal p53 can promote the transcription of ATP-binding cassette transporter A1 (ABCA1), a cholesterol efflux protein, thereby inhibiting the maturation of SREBPs precursors. The deletion of p53 leads to decreased expression of ABCA1 and upregulated expression of SREBP-1 62. Additionally, sustained activation of protein kinase B in liver cancer phosphorylates phosphoenolpyruvate carboxykinase 1 (PCK1) in the cytoplasm, and phosphorylated PCK1 promotes SREBP-1 to leave the endoplasmic reticulum and activate SREBP-1, thereby promoting tumor growth 63.

## Mechanism of TCM in regulating lipid metabolism

### TCM regulates fatty acid metabolism

It has been concluded in the above chapters that increased expression of ACLY, ACC, and FASN is the main manifestation of the reprogramming of lipid metabolism in tumor cells. Honeysuckle is a well-known TCM that has been widely used for several years. Its extract has been reported to inhibit the expression of ACLY and ACC1 64. In addition, Qingfei oral liquid, a TCM formulation with clinically proven anti-inflammatory properties, decreases ACLY expression by activating AKT signaling, thereby controlling fatty acid synthesis 65. These results indicate that the extracts or formulations of TCM could regulate the activity of ACLY and affect the synthesis of fatty acids.

Concerning ACC, Dang et al. found that Ling-gui-zhu-gan decoction markedly inhibited the activity of ACC, SREBP-1, and HMGCR, resulting in decreased lipid synthesis in the liver 66. Another Chinese medicine, Jinlida, ameliorates high-fat diet-induced insulin resistance in rats by reducing lipid accumulation and increasing AMPK and ACC phosphorylation in skeletal muscles 67. In addition, Polygonum multiflorum 68, Abrus mollis 69, danthron 70 and other TCM have been reported to inhibit the expression of ACC and FASN to regulate lipid metabolism. Therefore, TCM can regulate lipid metabolism by regulating these three key enzymes.

### TCM regulates cholesterol metabolism

#### Inhibition of cholesterol absorption in the intestine

Cholesterol is an important component of cell membranes and can be obtained through resynthesis from acetyl-CoA or from diet. There is a positive correlation between circulating low-density lipoprotein cholesterol (LDL-C) levels, which can damage arteries and cholesterol absorption. Studies have shown that TCM reduces cholesterol absorption. Water-soluble polysaccharides (WSP) from Cassia seeds bind to bile acids and reduce the amount of absorbable cholesterol 71. In addition, Wang et al. determined that berberine (BBR), a principal bioactive compound in Coptis chinensis and many other medicinal plants, decreases cholesterol levels in rats through multiple mechanisms, including the inhibition of cholesterol absorption 72. In the process of exploring this mechanism, the role of acetyl-CoA cholesterol acyltransferase (ACAT) (an enzyme that catalyzes cholesterol esterification in the cell and accelerates intestinal absorption) in mediating the regulation of cholesterol absorption by TCM has attracted the attention of researchers 73. Hawthorn (Crataegus pinnatifida) is an edible fruit used in Chinese medicine to lower blood lipid levels. Interestingly, Lin et al. found that hawthorn extract inhibited ACAT activity in Caco-2 cells. They further constructed an animal model demonstrating that triterpenic acids present in hawthorn extract reduced plasma cholesterol by inhibiting intestinal ACAT activity in hamsters 74.

#### Inhibition of endogenous cholesterol synthesis

Only one-third of cholesterol in the body comes from the food supply, with the rest coming from endogenous synthesis. Moriarty et al. evaluated the effect of Xuezhikang (XZK), an extract of fermented red yeast rice with lipid-lowering properties, on blood lipids in subjects with dyslipidemia but without coronary heart disease. The results of this multicenter, randomized, placebo-controlled study showed that daily administration of XZK 1200 mg and 2400 mg for 4-12 weeks resulted in statistically significant and clinically meaningful decrease in non-HDL-C and LDL-C levels compared to placebo 75. Notably, XZK contains a naturally occurring statin (monacolin K) that is identical to lovastatin. Importantly, statins reduce intracellular cholesterol synthesis primarily by competitively inhibiting HMGCR 76. These results suggested that XZK can restrict endogenous cholesterol synthesis by inhibiting HMGCR activity.

#### Promotion of cholesterol excretion in the liver

**Figure 1:**
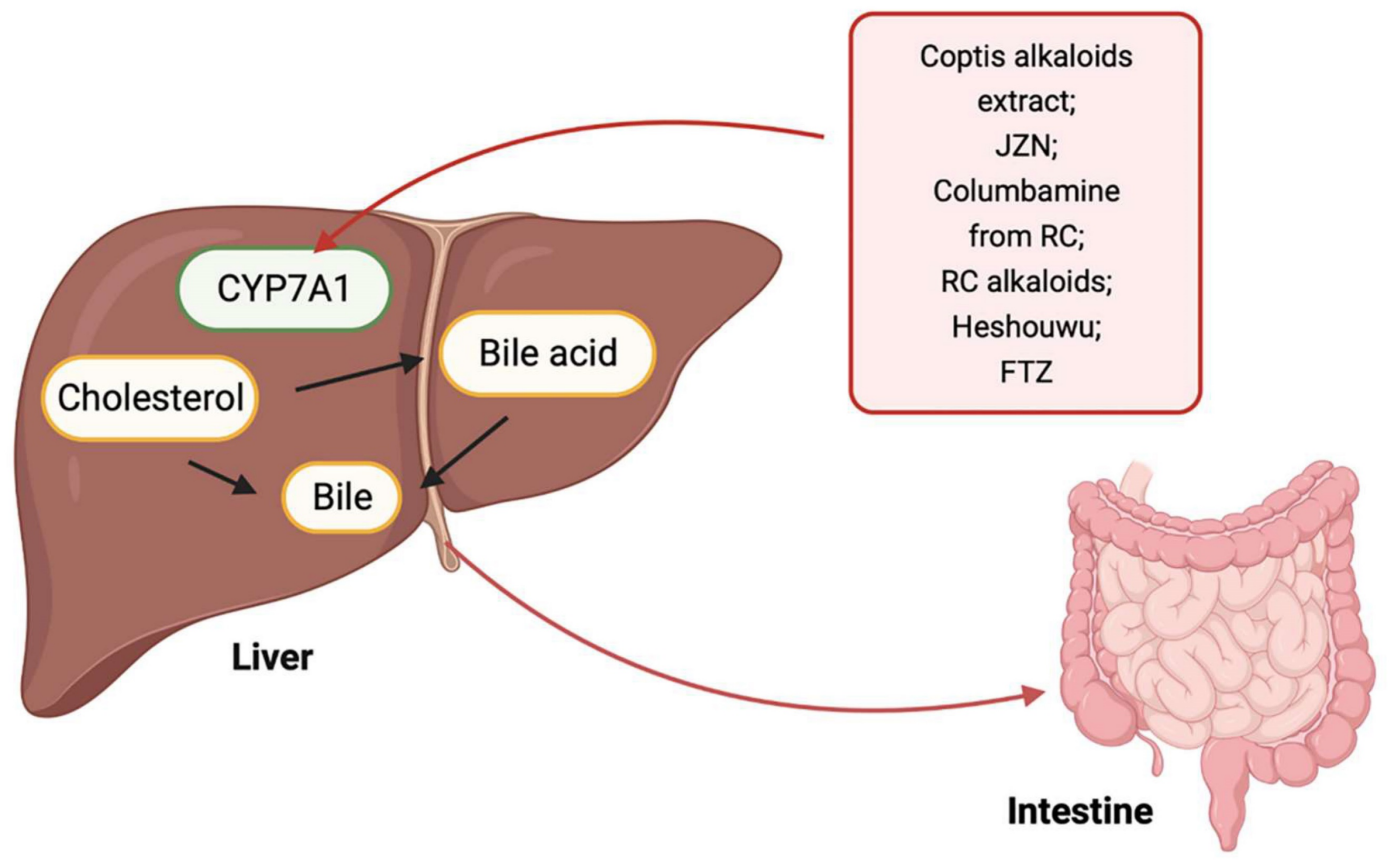
** Promotion of cholesterol excretion in the liver by TCM.** TCM including coptis alkaloids extract, JZN, Columbamine from RC, RC alkaloids, Heshouwu, and FTZ regulates the activity of CYP7A1 to promote the excretion of liver cholesterol into the intestine.

Cholesterol that is transported into the liver and endogenously synthesized is lost from the body via biliary secretion after conversion to bile acids. Cholesterol 7-alpha hydroxylase (CYP7A1) is the first rate-limiting enzyme in the neutral pathway of bile acid synthesis and is the main route for cholesterol removal from the body 77. An increasing number of studies have explored the role of TCM in promoting cholesterol excretion by the liver. For example, the coptis alkaloids extract, Jiang-Zhi-Ning (JZN), columbamine from Rhizoma coptidis (RC), RC alkaloids, and Heshouwu, regulate lipids associated with increased cholesterol conversion into bile acids by upregulating CYP7A1 mRNA level 78-82. In addition to the above Chinese herbal extracts or monomers, which can affect the excretion of cholesterol in liver by regulating the activity of CYP7A1, Chinese herbal compounds also play a role. Guo et al. developed Fufang Zhenzhu Tiao Zhi (FTZ), which comprises eight types of quality-maintaining Chinese herbs. By upregulating the expression and activity of the CYP7A1 gene, FTZ promotes the transformation of cholesterol into bile acids, thus reducing serum cholesterol in hyperlipidemic rats 83. However, the specific components that play a role in this compound are unclear and require further analysis. This mechanism is illustrated in Fig. 1.

## TCM regulates tumor lipid metabolism

Upregulation of FA anabolism is an important characteristic of tumor cell metabolism. Previous reviews have indicated that natural products derived from TCM suppress fatty acid biosynthetic pathways by targeting metabolic enzymes and are regarded as promising inhibitors for cancer treatment. Fatty acid-binding protein (FABP), an intracellular fatty acid transporter, is upregulated in many tumors. Liu et al. 84 isolated the novel natural triterpene GL22 from Ganoderma leucocontextum and showed that GL22 significantly inhibits the growth of liver cancer cell lines and tumor xenografts in vivo. Importantly, this study demonstrated that GL22 treatment decreased the expression of FABPs, which likely underlies the loss of cardiolipin, mitochondrial dysfunction, and cell death 84. In addition, Ganoderma tsugae (GT) has been reported to reduce the levels of fatty acids and lipids in prostate cancer cells by inhibiting the expression of SREBP-1, a key transcriptional regulator controlling lipogenesis, thereby inhibiting the growth of prostate cancer cells 85. In addition, oridonin, a diterpenoid isolated from Rabdosia rubescens 86 and Zhiheshouwu 87, has been reported to interfere with SREBP-1. Oridonin reduced the expression of SREBP-1 mRNA and protein in colorectal cancer cells, whereas Zhiheshouwu extract reduced fatty acid production via inhibiting SREBP-1 and its downstream factor stearyl-CoA dehydrogenase1 (SCD1) in hepatocellular carcinoma (HCC) cells thereby affecting fatty acid formation in tumors 86, 87. As a key enzyme in fatty acid synthesis, FASN plays an important role in tumor progression. Quercetin induced apoptosis of human HCC cells by inhibiting FASN activity and downregulating FASN expression 88. This indicates that TCM can inhibit tumor growth by interfering with FASN activity and affecting fatty acid production. Identifying potential targets for intervention may help improve efficacy and avoid drug resistance. Lin et al. found that in human epidermal growth factor receptor 2 (HER2)-overexpressed BC cells, the AKT/mTOR pathway mediated the inhibition of FASN expression by Osthole 89. In another study, demethoxycurcumin (DMC) derived from the rhizomes of turmeric decreased the activity and/or expression of FASN through AMPK activation in triple-negative breast cancer (TNBC) cells 90.

**Figure 2:**
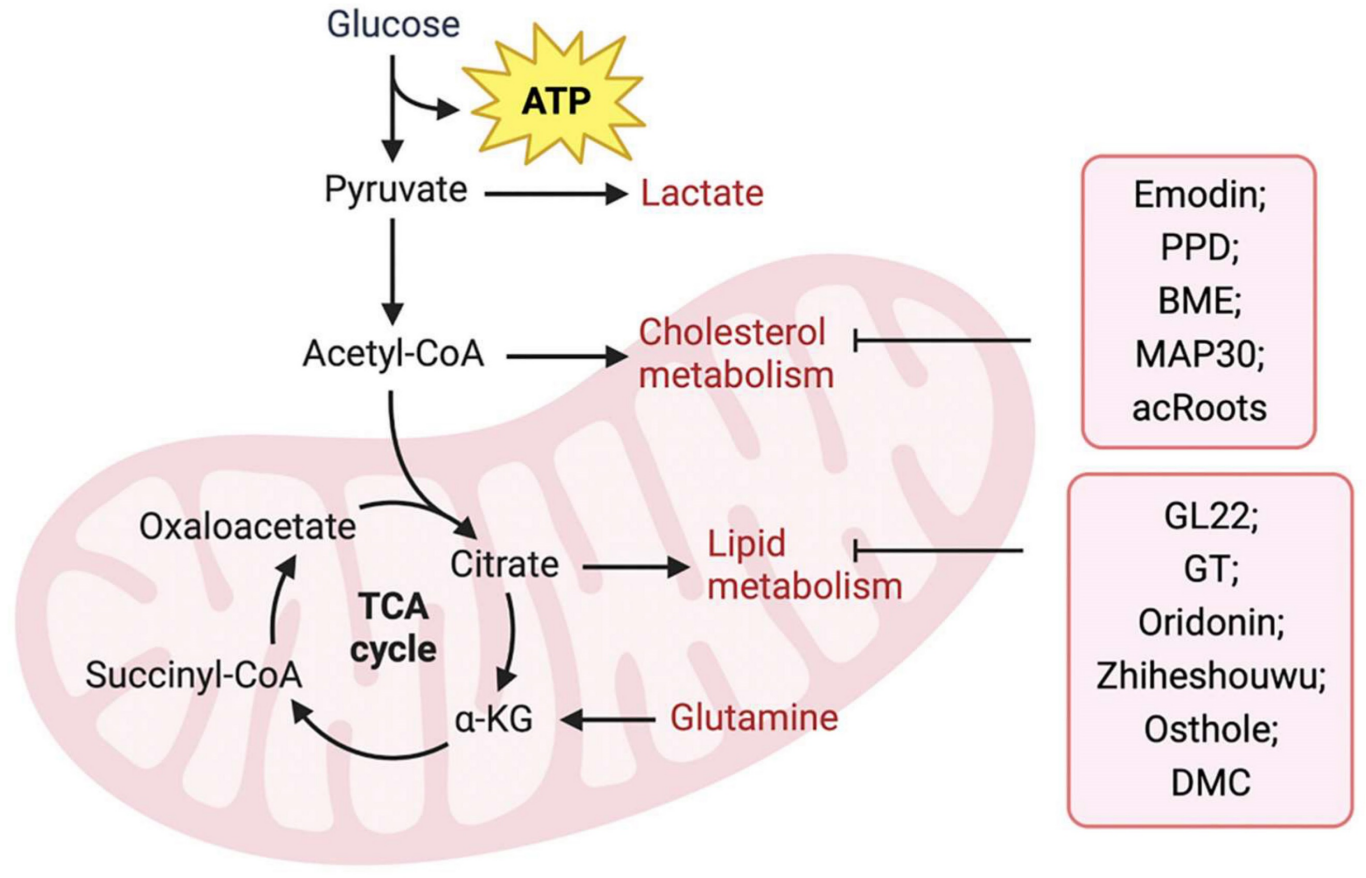
** TCM regulates tumor lipid metabolism.** TCM including Emodin, PPD, BME, MAP30, and acRoots, inhibits tumor progression by inhibiting fatty acid metabolism, while GL22, GT, Oridonin, Zhiheshouwu, Osthole, and DMC, inhibit tumor progression by inhibiting cholesterol metabolism in tumor cells.

Cholesterol metabolism is vital for the survival and growth of cancer cells (Figure 2). Emodin, an active component of Chinese herbs, sensitizes HCC cells to the anticancer effects of sorafenib by suppressing cholesterol metabolism. Mechanistically, emodin inhibits the sterol regulatory SREBP-2 transcriptional activity, which suppresses cholesterol biosynthesis and AKT signaling 91. In addition, TCM can affect tumor progression by regulating ACAT. Protopanaxadiol (PPD), a ginseng metabolite generated by gut bacteria, was shown to inhibit FASN and ACAT-2 expression, thereby inducing colorectal cancer cell death 92. In another study, Shim et al. showed that bitter melon extract (BME) treatment inhibited ACAT-1 expression in TNBC cells and reduced tumor growth in TNBC mammospheres implanted into NOD scid gamma mouse (NSG) mice 93. Another BME, momordica anti-HIV protein (MAP30), inhibits ovarian cancer cell progression by reducing glucose transporter (GLUT)-1/3 mediated glucose uptake, lipogenesis, and lipid droplet formation 94. Notably, Actinidia chinensis Planch root (acRoots) extract has been used to treat various types of cancers. A previous study indicated that it inhibits human HCC proliferation by reducing LDL uptake and intracellular cholesterol levels via reducing the expression of LDL receptor. However, the specific mechanism remains unclear and requires further investigation 95. Detailed information regarding the effects of natural products on lipid metabolism is summarized in Table 1.

## Conclusion and prospects

Reprogramming of lipid metabolism is an important feature of tumor cells. It is essential to explore safer and more effective antitumor treatment strategies by further identifying the dysregulated metabolic processes in tumor cells and understanding the molecular mechanisms related to metabolic reprogramming. Recent studies have shown that single-target inhibitors targeting the reprogramming pathway of lipid metabolism have not achieved ideal efficacy. With increasing studies on antitumor activities of TCM, it has been proven that TCM can effectively intervene in tumor metabolism, inhibit tumor cell proliferation, and promote tumor cell apoptosis through multiple targets and approaches. Combining TCM with current cancer treatment methods may provide ideas and programs for more effective clinical treatment of cancer.

However, most existing studies have focused on the regulation of tumor lipid metabolism by TCM monomers and their mechanisms, and relatively few studies have focused on the regulation of tumor metabolism by TCM compounds. TCM formulas have the advantage of being multi-component and multi-target, showing outstanding efficacy in clinical tumor treatment. However, complex drug compositions and various influencing factors may lead to difficulties in research. Therefore, a discussion on the mechanism of TCM formula intervention in tumor lipid metabolism reprogramming may be a direction for future research, which may better explain the scientific nature and reliability of TCM compound therapy for tumors.

## Figures and Tables

**Table 1:** Regulation of TCM and its bioactive compounds on lipid metabolism

Bioactive compounds	Chinese herbs	Cancer cells	Potential mechanisms	Ref.
GL22	Ganoderma leucocontextum	Liver cancer cells	Decreasing the expression of FABPs	84
/	GT	Prostate cancer cells	Inhibiting the expression of SREBP-1	85
Oridonin	Rabdosia rubescen	Colorectal cancer cells	Inhibiting the expression of SREBP-1	86
/	Zhiheshouwu	HCC cells	Inhibiting SREBP-1 and its downstream factor SCD1	87
/	Quercetin	HCC cells	Inhibiting FASN activity and downregulating FASN expression	88
Osthole	Cnidium monnieri (L.) Cusson	Breast cancer cells	Inhibiting FASN expression	89
DMC	Rhizomes of turmeric	TNBC cells	Inhibiting FASN expression	90
Emodin	/	HCC cells	Regulating the transcriptional activity of SREBP-2	91
Protopanaxadiol	Ginseng	Colorectal cancer cells	Inhibiting the expression of FASN and ACAT-2	92
BME	Bitter melon	TNBC cells	Inhibiting ACAT-1 expression	93
MAP30	Bitter melon	Ovarian cancer cells	Reducing GLUT-1/3 expression	94
acRoots extract	acRoots	HCC cells	Reducing LDL uptake and intracellular cholesterol levels	95

## Abbreviations

PI3K: phosphatidylinositol 3 kinase
AKT: protein kinase B
AMPK: adenosine monophosphate-activated protein kinase
mTOR: mammalian target of Rapamycin
ATP: adenosine-triphosphate
TCM: traditional Chinese Medicine
ACLY: ATP-citrate lyase
FASN: fatty acid synthase
HMGCR: 3-hydroxy-3-methylglutaryl-CoA reductase
BC: breast cancer
DCIS: ductal carcinoma in situ
FAO: fatty acid oxidation
CPT1: carnitine palmitoyl transferase 1
SREBP-1: sterol regulatory element-binding proteins-1
LXRs: liver X receptors
ABCA1: ATP-binding cassette transporter A1
PCK1: phosphoenolpyruvate carboxykinase 1 in cytoplasm
LDL-C: low density lipoprotein cholesterol
WSP: Water-soluble polysaccharides
BBR: berberine
ACAT: acetyl-coA cholesterol acyltransferase
XZK: Xuezhikang
CYP7A1: cholesterol 7-alpha hydroxylase
RC: RhizomaCoptidis
FTZ: Fufang Zhenzhu Tiao Zhi
FABP: fatty acid binding protein
GT: Ganoderma tsugae
SCD1: stearyl-CoA dehydrogenase1
HCC: hepatocellular carcinoma
DMC: demethoxycurcumin
PPD: protopanaxadiol
BME: bitter melon extract
acRoots: actinidia chinensis Planch root
